# House dust mites in three contrasting climatic regions of Saudi Arabia

**DOI:** 10.1007/s10493-026-01123-0

**Published:** 2026-02-27

**Authors:** Riyadh Hussain S. Aeban, Medjedline Hani, Henk R. Braig, M. Alejandra Perotti

**Affiliations:** 1 Ecology and Evolutionary Biology, School of Biological Sciences, University of Reading, Whiteknights Campus, HLS Bld, Reading, RG6 6AS UK; 2 Department of Medical Molecular Laboratory, Security Forces Hospital Makkah, Makkah, Saudi Arabia; 3 Institute and Museum of Natural Sciences (IMCN), National University of San Juan (UNSJ), San Juan, Argentina

**Keywords:** Acari, House dust mites, Allergens, Biodiversity, Pyroglyphidae, Cheyletidae, Dermatophagoides, Oribatida, HDM, Arabian Peninsula

## Abstract

Just a handful of mite species are suspected inhabiting the dust of houses within arid areas of the Arabian Peninsula. In this study, we identified and compared the house dust mite diversity in three different climatic areas within Saudi Arabia. Sampling was carried out over one year and two months, with samples collected in the desert, the coast, and in the highlands. It reviews all known mites of house dust of Saudi Arabia. A total of 998 mites were collected belonging to 23 species in 14 families. Pyroglyphidae (Astigmata) were the most abundant. The interaction among pyroglyphid species in the climatic regions was analysed. While variation in abundance of Pyroglyphidae among regions was weak, there was a strong desert region *Cheyletus eruditus*–*Dermatophagoides farinae* association, perhaps explaining that *D. farinae* populations from the desert have become *C. eruditus’* favourite prey. Another interesting association is that of Pyroglyphidae with Acaridae. *D. pteronyssinus* shows significant numerical dominance in the highlands. A newly discovered species of *Dermatophagoides* did not interact with any of the other 2 species and was restricted to the coastal region. *Dermatophagoides* n. sp. and *Cheletopsis* sp. can be used as markers of location in forensic analyses (provenance), based on their unique association with the coast and desert, respectively. A new species of *Dermatophagoides* has been identified. Five species are new to Saudi Arabia and the Arabian Peninsula including house dust, ten species are new to the house dust mite fauna of Saudi Arabia and the Arabian Peninsula but have been recorded as storage mites, totalling 15 new house dust mites for the Arabian Peninsula. Two species are new to house dust in Saudi Arabia, but already known from house dust in Kuwait. Of the fifteen new house dust mites for the Arabian Peninsula, five species are new to the dust mite fauna worldwide: Prostigmata: *Cheletopsis* sp. (Cheyletidae); Mesostigmata: *Proctolaelaps pygmaeus* (Melicharidae), *Macrocheles muscaedomesticae* (Macrochelidae); and Oribatida: *Oribatula tibialis* (Oribatulidae), and *Ceratozetes* sp. (Ceratozetidae).

**Supplementary Information:**

The online version contains supplementary material available at 10.1007/s10493-026-01123-0.

## Introduction

Domestic mites (Acari) inhabit human dwellings, predominantly as house dust or pulvicolous mites, as stored products mites, and as mites of pet animals and house mice (Ogata et al. 1980; Faccini and Coutinho 1987; Hagstrum et al. 2013b; Abdel-Rahman et al. 2020; Kontschán et al. 2022; Kamezaxi et al. 2024; Kontschán and Hornok 2025; Yu et al. 2025). The mites associated with human habitation are being studied extensively and from almost every corner of the world with recent additions from, for example, Egypt (Bakr et al. 2022; Ibrahim et al. 2022), India (Saw et al. 2024), Iran (Zare et al. 2021), Ireland (Aljohani et al. 2024), Japan (Kamezaki et al. 2022), Panama (Lezcano et al. 2020), the Philippines (Corpuz-Raros 2022), Poland (Solarz et al. 2021), or Türkiye (Akdemir et al. 2023; Aykut 2024). House dust mites belonging mainly to the family Pyroglyphidae are feeding on mould (fungi) and, among other things, on human and pet animal skin flakes. The most ubiquitous species are *Dermatophagoides farinae* Hughes, 1961, *D. pteronyssinus* (Trouessart, 1897), *D. evansi* Fain, Hughes & Johnston, 1967, and *Euroglyphus maynei* (Cooreman, 1950). Storage mites include mites that feed on the stored products such as grain, and fungivore and other detritivore mites. The most prominent families are Acaridae, Echymyopodidae, Glycyphagidae, Chortoglyphidae and Suidassidae represented by *Acarus siro* Linnaeus, 1758, *Thyreophagus entomophagus* (Laboulbène & Robin, 1862), *Tyrophagus putrescentiae* (Schrank 1781); *Blomia tropicalis* Van Bronswijk, De Cock and Oshima 1973; *Glycyphagus domesticus* (de Geer, 1778), *Lepidoglyphus destructor* (Schrank 1781); *Chortoglyphus arcuatus* (Troupeau, 1879); *Suidasia medanensis* Oudemans, 1924, and *S. pontifica* Oudemans, 1905 (Wharton 1976; Malainual et al. 1995; Petrova and Zheltikova 2000; Takaoka 2000; Pike and Wickens 2008; Yu et al. 2015; Podder et al. 2021; Solarz et al. 2021; Acuna-Cantillo et al. 2024; Aykut 2024). The concentration of storage mites might be underestimated compared to house dust mites (Reboux et al. 2019). There is some overlap between the two groupings, for example, *B. tropicalis* is often also considered a house dust mite. In animal studies, storage mites might produce greater inflammation of the lungs than house dust mites (Kim et al. 2024). Dust mites only by appearance, which are not associated with houses or storage such as date palm dust mite, *Oligonychus afrasiaticus* (McGregor, 1939), are not covered here.

The mite fauna of indoors can be quite rich. Oribatida (soil mites) can be brought inside houses (Chen et al. 2023), members of the Tetranychidae (Prostigmata) can also be found associated with ornamental plants. All these mites attract their natural predators to come indoors as well, particularly from the family Cheyletidae with *Cheyletus malaccensis* (Oudemans, 1903) and *C. eruditus* (Schrank 1781) being most widely distributed in houses (Zhou et al. 2022). Ticks only occur together with their household pets, the study of their development under domestic conditions has just begun (Buczek et al. 2023; Shinohara et al. 2023).

A new build is practically free of mites (Ouchi et al. 1976; Crowther et al. 2000). Humans introduce house dust mites mostly with their clothing (Perotti and Braig 2009; Clarke et al. 2015). When houses become uninhabited, the dust mites start to disappear too but are replaced by other species like *Tyrophagus putrescentiae*, species of Glycyphagidae, Tarsonemidae, Cheyletidae and Oribatida, as this has been seen in houses uninhabited for 6–7 years in Fukushima, Japan; where the total amount of mites stayed roughly the same (Shinohara et al. 2023). The species more dominant in uninhabited houses are known from dust of houses built in the past more open to the environment.

Domestic mites may pose health risks (Cui 2014; Hubert et al. 2018). Most dust mites are allergenic, but only a fraction of the 280 or so storage mite species are major sources of allergens (Hagstrum et al. 2013a; Fernández-Caldas et al. 2014). Micro- and nanoplastics increase the allergenic potential of mite allergens (Wang et al. 2025; Wu and Yang 2025). The indoor mite fauna is also of increasing forensic interest (Perotti et al. 2009; Solarz 2009; Frost et al. 2010; Çakan et al. 2015; Perotti and Braig 2019; Solarz et al. 2022).

The fast adaptation to the peculiarities of human household environments is reflected in the genomes of house dust and storage mites (Xiong et al. 2022; Hubert et al. 2023; Klimov et al. 2024; Vidal-Quist et al. 2025). Humidity and temperature are crucial parameters in the distribution of domestic mites. House dust mites mainly absorb water from humid air. The active breeding zone has been defined for a relative humidity of 55 to 75% at 15 to 35 ˚C. (Arlian 1992; Pei et al. 2020; Vackova et al. 2021, 2023). Eggs of *D. pteronyssinus* withstand for 80% 40 ˚C dry heat, but for direct sunlight and 50 ˚C dry heat, the thermal death point occurs at 3 and 5 h, and for 60 and 70 ˚C, at 30 min (Mahakittikun et al. 2011). This impacts domestic mites in desertic regions like the Arabian Peninsula.

**Fig. 1:**
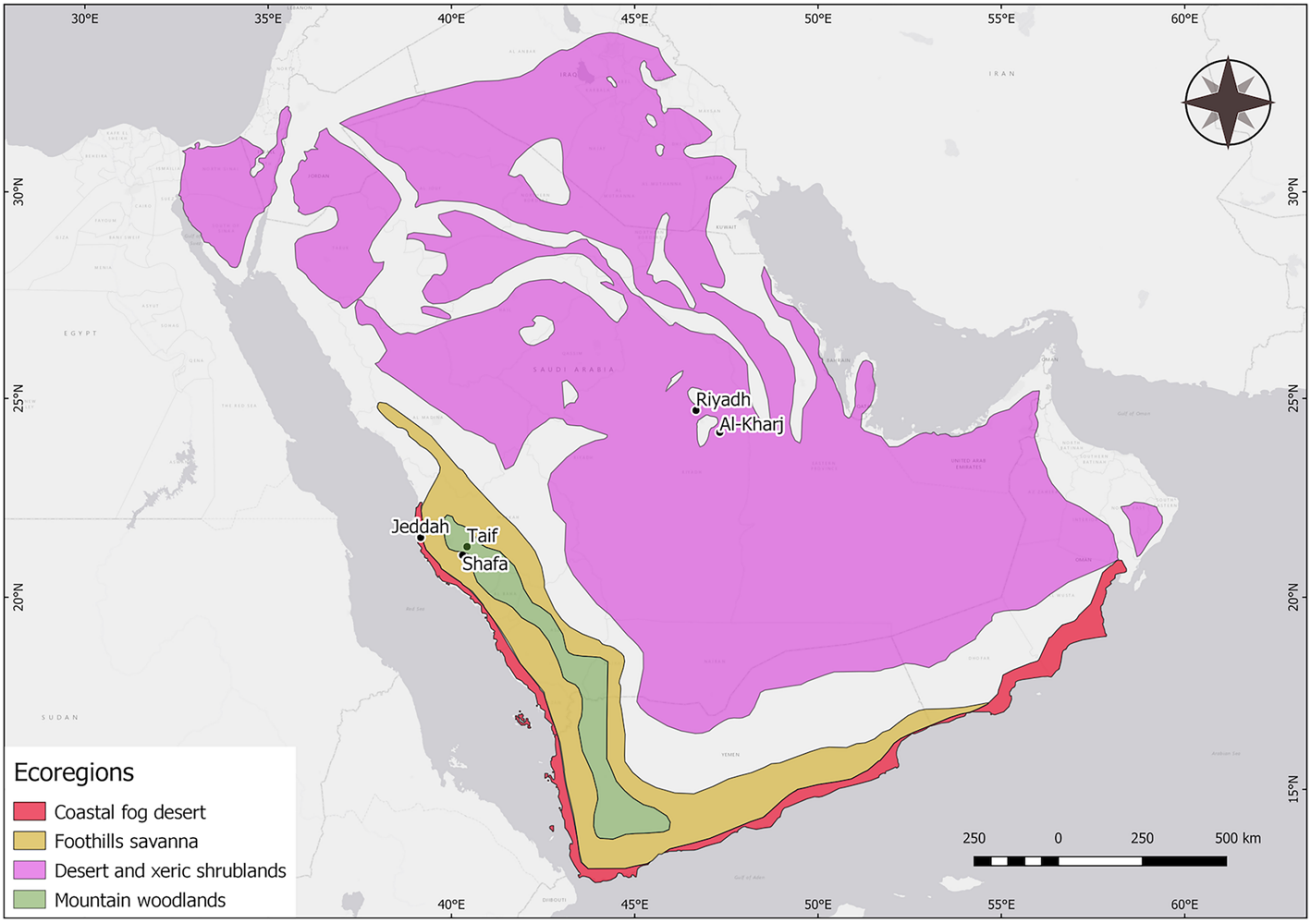
Five cities in the three climatic regions, the Desert composed of the Arabian desert and East Sahero-Arabian xeric shrublands belonging to the Palearctic realm; the Coast being the Arabian Peninsula coastal fog desert; and the Highlands made up of the Southwestern Arabian foothills savanna, and the Southwestern Arabian montane woodlands (mountain woodlands), all belonging to the Afrotropical realm

In this work we were interested in exploring the diversity and abundance of the domestic mite fauna in major Saudi cities in three ecoregions of the Arabian Peninsula, the desert and xeric shrublands with Riyadh and Al-Kharj, the coastal fog desert with Jeddah, and the highlands which themselves can be subdivided in the foothills savanna with Shafa and the montane woodlands with Taif (Fig. 1).

The importance of house dust mites for producing aeroallergens in respiratory diseases and disorders such as allergic rhinitis, asthma, asthmatic flare-ups, cat allergen sensitization and atopic dermatitis is often high or even the highest across the climate ranges of Saudi Arabia and the Arabian Peninsula (Table 1). This importance is measured against other indoor allergens like cockroaches and pet dander (cats, dogs), and outdoor allergens such as plants (prickly saltwort, lamb’s quarters, ragweed, pigweed, Russian thistle), grasses (Bermuda grass, Timothy grass, rye), trees (several mesquites), and moulds, *Alternaria* and *Aspergillus* species. These studies on aeroallergens are mainly based on *Dermatophagoides farinae* and *D. pteronyssinus*, because most commercial test kits used in literature cover only these two mite species (Khurram et al. 2025). The diversity of the actual house dust mites has been little researched. Studies have been limited to two locations on the coast only, Dammam (Al-Qurashi 2006) and Jeddah (Edrees 2008, 2009, 2010, 2012). There are no records of house dust mites in the desert regions or in the highlands. In this work we explored the diversity and abundance of the House Dust Mite (HDM) fauna in three climatic regions of Saudi Arabia.

**Table 1:** Importance of house dust mite allergens in Saudi Arabia and in the Arabian Peninsula

Condition	Importance	Location
Allergic rhinitis		
Coast	high	Western SA (Badran et al. 2016); Bahrain (Hasan and Rizwan 2015) Oman (Al-Tamemi et al. 2008; Al-Abri et al. 2015); Qatar (Sattar et al. 2003; Zahraldin et al. 2021; Thalappil et al. 2023); UAE (Sultan and Khalil 2012; Mahboub et al. 2014; Asad et al. 2015), children (Adham and Tawfik 2012; Al Sharif et al. 2017; Sharif et al. 2018)
	low to moderate	Jordan (Aburuz et al. 2011), Kuwait (Dowaisan et al. 2000; Ezeamuzie et al. 2000a; Al-Dowaisan et al. 2004); children (al Mousawi et al. 2009; Abal et al. 2010), UAE (Bener et al. 2002)
Desert	high	Riyadh (Almogren 2009)
	low to moderate	Riyadh (Al-Shalan et al. 1989; Zakzouk and Gad-El-Rab 1996), Najran (Alqahtani 2020), children (Alqahtani 2016); Qassim (Alrasheedi et al. 2023a)
Highland	high	Abda children (Asseri et al. 2025) Yemen (Al-Mehdar et al. 2025)
	moderate	Jazan (Zaki et al. 2019)
Nation	low	Abha (Al-Ghamdi et al. 2022)
	highest	Saudi Arabia (Emara et al. 2012; Almehizia et al. 2019)
Asthma		
Coast	high	Jazan (Alhazmi et al. 2025); Jeddah (Al-Frayh et al. 1997; Koshak 2006), Al-Khobar (Al-Nahdi and Al-Quorain 1987), children (Al Khater 2017), Makkah (Tayeb 2015), children (Al-Frayh et al. 1992); Oman (Al-Tamemi et al. 2008); Qatar (Sattar et al. 2003; Zahraldin et al. 2021; Thalappil et al. 2023); UAE (Sultan and Khalil 2012; Asad et al. 2015), children (Al-Aani and Alhammadi 2015; Al Sharif et al. 2017; Sharif et al. 2018)
	low to moderate	Kuwait (Ezeamuzie et al. 2000a, b; Khadadah et al. 2000a, b; al Mousawi et al. 2001); children (Hijazi et al. 2002; al Mousawi et al. 2009; Abal et al. 2010); UAE (Bener et al. 2002)
Desert	high	Riyadh (Almogren 2009)
	low to moderate	Riyadh (Al-Frayh et al. 1997; Gad-El-Rab 1999), children (Al-Frayh et al. 1992); Najran (Alqahtani 2020), children (Alqahtani 2016)
Highland	high	Abhar (Al-Frayh et al. 1997), Aseer (Al-Ghamdi et al. 2019), Jazan (Zaki et al. 2019) Yemen (Al-Mehdar et al. 2025)
	moderate	Tabuk (Al-Dusari et al. 1997)
Nation	highest	Saudi Arabia (Emara et al. 2012)
Asthma flair ups		
Desert	highest	Qassim (Alrasheedi et al. 2023b)
Cat allergen sensitization		
Coast	high	Jeddah (Tayeb 2020)
Atopic dermatitis		
Coast	highest	UAE children (Adham et al. 2011)
Desert	little	Najran (Alqahtani 2016, 2020)
Highland	moderate	Tabuk (Al-Dusari et al. 1997)

## Methods

### General information and sampling

House dust samples were collected over a period of one year and two months, starting in December 2016 and finishing in January 2018. The dust samples were provided by 5 households, each of them from each of the 5 Saudi cities: Al-Kharj, Jeddah, Riyadh, Shafa, and Taif; therefore, a total of 25 sampling sites. For *Dermatophagoides* spp. repeated samplings were conducted (x2) totalling 50 samples (Table S2); a 1 st sampling in 2017 was only on *Dermatophagoides* spp while 2nd sampling attempt in 2018 included all mites.

Desert: The city of Riyadh, 24°38′N 46°43′E, is at an elevation of 600 m situated in the an-Nafud desert on the eastern part of the Najd plateau, and Al-Kharj (24°08′54.0″ N 47°18′18.0″E) is an oasis southeast of Riyadh, both in Riyadh Province. Climate is hot dry with desert subzone. They belong to the Arabian desert in the Palearctic realm. Low relative humidity with a trough as low as 10% in June and a peak of up to 50% in December to January (Meteo Portal 2017).

Coast: Jeddah, 21°32′36″N 39°10′22″E, Makkah Province, is a port city at the Red Sea. Climate is hot dry maritime desert. It belongs to the tip of the Northern tip of Arabian Peninsula coastal fog desert or xeric shrub ecoregion in the Afrotropical realm although it does not experience so much fog. Relative humidity around Jeddah can be very high, fluctuating between low 40 s in May to July to over 80% in October.

Highlands: Ash-Shafa, 21°04′10″N 40°18′43″E, is at an elevation of 2,240 m and Taif, 21°16′30.34″N 40°24′22.16″E, is at 1,880 m are on the slopes of the Hijaz Mountains that belong to the Sarat Mountains; they both are also in Makkah Province. These two cities are close together, yet, Shafa, despite its elevation, is considered part of the foothills savanna while Taif is part of the montane woodlands ecoregion in the Afrotropical realm. Climate is sub-tropical with a Mediterranean subzone and a mountainous subtype. Relative humidity ranges between 20% in June and 60% in January.

### Processing of samples for their arthropod content

Dust samples were collected from human dwellings by their owners or inhabitants with the aid of their own vacuum cleaner devices, extracted from floors, carpets and rugs at a rate of 1 m^2^ per minute and for 5 min. Once the vacuuming process was completed, the dust collected from the 5 households (of each city) were transferred into jars containing 96% ethanol, followed by alcohol evaporation afterwards, all done by the Saudi Arabian 1 st author in its home institution (see authors affiliations). The samples were poured from the jars into open sealing bags contained in trays (so widely open) allowing slow evaporation for up to 3 days (to avoid shrinking and loss). The preserved/fixed arthropods into labelled sealed plastic bags were transported to the laboratory in UK for further analysis.

Once in the laboratory at the University of Reading, UK, the preserved dry specimens mixed with some dust particles were rehydrated in 70% alcohol (v/v, water/alcohol). For this, the bags were re-filled with 70% alcohol and allowed rehydration of specimens over night to avoid shrinking and potential loss. Then they were divided in subsamples to ease the examination under the stereomicroscope (Motic SMZ-171). All micro-arthropods and/or arthropod parts were separated for further identification. The extracted mite specimens were then permanently mounted on glass slides in Hoyer’s medium for microscopic examination (Krantz 1978). The material was deposited in the Forensic Acarology Collection at the University of Reading.

A Nikon Optiphot phase contrast microscope was utilised for identification of the specimens. Images were captured using a Motic Moticam 3 Plus (microscope digital camera). Accordingly, the following keys and descriptions were used for mite families and species identification (Fain 1967; Van Bronswijk et al. 1973; Van Bronswijk and De Cock 1973; Hughes 1976; Wharton 1976; Perez 1993; Colloff 1998; Mašán 2003; Krantz and Walter 2009; Michaud et al. 2012; Solarz 2012; Alatawi et al. 2018; Kamran et al. 2018). For the separation of *Dermatophagoides* species, the key of Hart and Fain (1988) was used, considering the epimeral plates (ventral), hysteronotal shield and middle dorsum, bursa copulatrix (females) and size (thickness) of legs 1 and 3 (males).

### Data analyses

Data were counts of individual mites per species, family, by city and region. Boxplots were built for mite counts allowing visualization of families or species and totals (StataCorp 2014). Kruskal-Wallis rank tests were used to compare species of Pyroglyphidae by region and in more detail, by city. In order to check for dependency between the mite species in the Pyroglyphidae (*Dermatophagoides*) and their locations, Pearson pairwise correlations and the Kendall rank correlation coefficient (Kendall’s τ b but no Effect size) were also estimated with an alpha of 0.05. These are non-parametric tests and do not depend on assumptions founded on the type of distribution.

A Generalized Linear Model (GLM) Poisson Regression based, Log likelihood, was applied to study the predator-prey interaction by region using (1) *Cheyletus eruditus* (HDM predator) as dependent variable, and *D. farinae*, *D. pteronyssinus*, *Dermatophagoides* n. sp. and *Blomia tropicalis* as independent variables; and (2) *Cheyletus eruditus* vs. Pyroglyphidae and Acaridae. In addition, a *t*-test for paired samples was conducted between Pyroglyphidae and *Cheyletus eruditus* to account for dependency of the data. Data from the remaining families were not statistically analysed due to the low counts in each family and the great number of zeros (0). In addition, indicator species analysis (IndVal) was applied on the three regions; for which *p*-values of indicators were run by 9999 random permutations (with an alpha of 0.05). The *p*-values were Bonferroni corrected. Graphs and statistical analyses were performed with StataCorp (2014) and PAST4 (Hammer et al. 2001; Hammer and Harper 2024).

Articles published in journals considered to be predatory are not referenced.

## Results and Discussion

### Diversity of house-dust mites

 Five hundred and seventy four specimens of allergy-causing *Dermatophagoides* and *Blomia* species (Pyroglyphidae, Echimyopodidae) have been collected (Tables S1 (sampling of 2018) and S2 (*Dermatophagoides* repeated sampling)). While mite allergens have been widely reported, no mites had been collected before in the desert and highlands.

A total of 998 mites were identified from 4 mite orders, Astigmata (considered a cohort within the Oribatida), Oribatida, Mesostigmata, and Prostigmata, 14 families, and 23 identified morpho-species (Tables S1 and S2).

A new species of *Dermatophagoides* (Pyroglyphidae) has been collected.

*Sancassania berlesei (*Acaridae), *Histiostoma feroniarum* (Histiostomatidae), *Suidasia pontifica* (Suidasiidae), *Cheletopsis* sp. (Cheyletidae) and *Ceratozetes* sp. (Ceratozetidae) are first records for Saudi Arabia and the Arabian Peninsula (Table 3).

*Glycyphagus domesticus* (Glycyphagidae), *Histiostoma* sp. not *H. feroniarum* (Histiostomatidae), *Cheyletus eruditus* (Cheyletidae), *Bryobia* sp. and *Eutetranychus* sp. (Tetranychidae), *Macrocheles muscaedomesticae* (Macrochelidae), *Proctolaelaps pygmaeus* (Melicharidae), *Scheloribates* sp. (Scheloribatidae), *Oribatula tibialis* (Oribatulidae), and *Scheloribates* sp. (Scheloribatidae) are first records for house dust in Saudi Arabia and the Arabian Peninsula (Table 3).

*Tetranychus* spp. (Tetranychidae) and *Stratiolaelaps miles* (Laelapididae) are new records for house dust in Saudi Arabia; they have been previously described for house dust in Kuwait (Table 3).

Among these, 5 species are new to the dust mite fauna worldwide: Prostigmata: *Cheletopsis* sp. (Cheyletidae); Mesostigmata: *Proctolaelaps pygmaeus* (Melicharidae), *Macrocheles muscaedomesticae* (Macrochelidae); and Oribatida: *Oribatula tibialis* (Oribatulidae), *Ceratozetes* sp. (Ceratozetidae) (Table 3).

A detailed discussion of the mites collected and the nature of the findings is presented after Table 3.

**Table 2:** Mite allergens and mites in the Arabian Peninsula

	Mite allergens in literature Saudi Arabia [% of sensitizations]a	Mite allergens in literature Arabian Peninsula [% of sensitizations]a	# Mites collected here in Saudi Arabia 1 st - 2nd*
Dermatophagoides			
*D. farinae*			
Coast	84% (Koshak 2006), 56% (Al-Frayh et al. 1997), 48% (Tayeb 2020), 36% (Hassan et al. 2013), 33% (Badran et al. 2016), 31% (Hasnain et al. 2012), 29% (Hasnain et al. 2012), 11% (Tayeb 2015); children 50% (Al-Frayh et al. 1992), 48% (Al Khater 2017).	Bahrain (Hasan and Rizwan 2015) Jordan 22% (Aburuz et al. 2011), 11% (Khasawneh et al. 2019), 8% (Al-Zayadneh et al. 2019), 2% (Khreesha et al. 2020); children 88% (Khreesha et al. 2020). Kuwait 39% (Ezeamuzie et al. 2000b), 32% (Dowaisan et al. 2000), 30% (Al-Dowaisan et al. 2004), 16% (Tubeilah and Luqman 1987). Oman 48% (Al-Tamemi et al. 2008). Qatar 39% (Thalappil et al. 2023), 37% (Sattar et al. 2003), children 29% (Zahraldin et al. 2021). UAE 42% (Hasnain et al. 2012), children and adults (Yousif et al. 2025); children 89% (Adham and Tawfik 2012), 75% (Adham et al. 2011), 37% (Al Sharif et al. 2017; Sharif et al. 2018), 18% (Lestringant et al. 1999), 16% (Al-Aani and Alhammadi 2015)	19–43
Desert	38% (Hasnain et al. 2012), 13% (Al-Frayh et al. 1997), 20% (Al-Shalan et al. 1989), 11% (Zakzouk and Gad-El-Rab 1996), 7% (Alqahtani 2020); 10% children (Al-Frayh et al. 1992; Alqahtani 2016).		5–11
Highlands	40% (Hasnain et al. 2012) 19% (Al-Frayh et al. 1997), 16% (Al-Ghamdi et al. 2019), 12% (Al-Ghamdi et al. 2022).	Yemen children and adults 10% (Al-Mehdar et al. 2025)	33–90
D. microceras			
Coast	_	Kuwait 45% (Ezeamuzie et al. 2000b), 39% (Dowaisan et al. 2000).	_
D. pteronyssinus			
Coast	87% (Koshak 2006), 47% (Tayeb 2020), 39% (Hasnain et al. 2012), 37% (Badran et al. 2016), 36% (Suliaman et al. 1997), 28% (Hasnain et al. 2012), 20% (Hassan et al. 2013), 18% (Tayeb 2015); children 45% (Al Khater 2017).	Bahrain 42% (Hasan and Rizwan 2015) Jordan 33% (Aburuz et al. 2011), 18% (Khreesha et al. 2020), 14% (Khasawneh et al. 2019), 12% (Al-Zayadneh et al. 2019); children 66% (Khreesha et al. 2020). Kuwait 54% (Hijazi et al. 2002), 53% (Ezeamuzie et al. 1997), 46% (Ezeamuzie et al. 2000b), 38% (Ezeamuzie et al. 2000a; Al-Dowaisan et al. 2004), 36% (Dowaisan et al. 2000), 29% (Khadadah et al. 2000b), 28% (Khadadah et al. 2000a), 23% (Ezeamuzie et al. 1997), 20% (Tubeilah and Luqman 1987), children 4% (al Mousawi et al. 2009). Oman 51% (Al-Tamemi et al. 2008). Qatar 50% (Thalappil et al. 2023), 42% (Sattar et al. 2003); children 38% (Zahraldin et al. 2021). UAE 46% (Hasnain et al. 2012), children and adults (Yousif et al. 2025), children 96% (Adham and Tawfik 2012), 75% (Adham et al. 2011), 37% (Al Sharif et al. 2017; Sharif et al. 2018), 18% (Lestringant et al. 1999), 14% (Al-Aani and Alhammadi 2015).	24–28
Desert	37% (Hasnain et al. 2012), 13% (Alqahtani 2020); children 15% (Alqahtani 2016).		5–7
Highlands	40% (Hasnain et al. 2012), 25% (Al-Frayh et al. 1997), 23% (Al-Ghamdi et al. 2019), 17% (Al-Ghamdi et al. 2022).	Yemen children and adults 10% (Al-Mehdar et al. 2025)	55–143
*Blomia tropicalis*			
Coast	17% (Hassan et al. 2013).	_	5 - --
Highlands		Yemen children and adults 4% (Al-Mehdar et al. 2025)	3 - --

^a^ Percentages of sensitizations refer in most but not all cases to patient cohorts of allergy clinics; these percentages are only indicative; they only make sense in the specific context of each study, and in some comparisons between mite species. The last column enumerates the number of mites of these species found in the various climate regions in this survey. *(2018) Second or repeated sampling of D. farinae * D. pteronyssinus* mites only

**Table 3:** House Dust Mite Species collected in Saudi Arabia, with related literature

	House dust Saudi Arabia	Apart from house dust Saudi Arabia	House dust Arabian Peninsula
Astigmata Pyroglyphidae			
*Dermatophagoides farinae*	C 62^a^; Dammam (Al-Qurashi 2006), Jeddah (Edrees 2008, 2009, 2010, 2012) D 16, H 123	Storage Jeddah	Kuwait (Gamal-Eddin and Hamad 1992)
*D. pteronyssinus*	C 52; Jeddah (Edrees 2008, 2009, 2010, 2012) D 12, H 198		Kuwait (Gamal-Eddin et al. 1985; Gamal-Eddin and Hamad 1992)
*Dermatophagoides* n. sp.	C 104, D 0, H 0 New species	New species	New species
Acaridae			
*Aleuroglyphus ovatus*	C 11; Jeddah (Edrees 2008, 2009, 2012) D 1, H 3		
*Acarus siro*	C 1; Jeddah (Edrees 2009; Edrees 2012) D 2, H 26		
*Sancassania berlesei*	C 2, D 0, H 0 First record		First record
*Tyrophagus putrescentiae*	C 15; Jeddah (Edrees 2009; Edrees 2012) D 0, H 28	Hail (Al-Shemmary 2016)	Kuwait (Gamal-Eddin et al. 1985; Gamal-Eddin and Hamad 1992)
Carpoglyphidae			
*Carpoglyphus lactis*	C Jeddah (Edrees 2012)	Jeddah	
Echimyopodidae			
*Blomia freemani*	C Jeddah (Edrees 2009, 2012)		
*B. tropicalis*	C 5; Jeddah (Edrees 2008, 2009, 2010, 2012) D 0, H 3		Kuwait (Gamal-Eddin and Hamad 1992)
Glycyphagidae			
*Glycyphagus domesticus*	C 2, D 1, H 5 First record	Riyadh (Al-Khalifa and Bayoumi 1983)	First record
Histiostomatidae			
*Histiostoma feroniarum*	C 6, D 0, H 1 First record	Likely first record	First record
*Histiostoma* sp.	C 0, D 0, H 5 First record	Majmaah (Mashaly et al. 2011)	First record
Suidasiidae			
*Suidasia pontifica*	C 7, D 0, H 6 First record		First record
*S. nesbitti*	C Jeddah (Edrees 2008, 2009, 2012)		
Prostigmata			
Acarophenacidae			
*Acarophenax tribolii*		Storage Jeddah (Al-Nasser 2011)	
Caligonellidae		Storage Riyadh (Rostom 1993)	
Cheyletidae			
*Acaropsella volgini*		Storage Dammam, Riyadh (Rostom 1993)	
*Acaropsis sollers*		Storage Dammam Riyadh (Rostom 1993)	
*Cheyletus eruditus*	C 29, D 51, H 37 First record	Riyadh (Al-Youssif and Soliman 1979; El-Bahrawy and Al-Dakhil 1993)	First record
*C. malaccensis*	C Jeddah (Edrees 2008, 2009, 2012)	Storage Dammam (Rostom 1993), Jeddah; Hail (Al-Shemmary 2016), Riyadh (Al-Youssif and Soliman 1979)	Kuwait (Gamal-Eddin and Hamad 1992)
*Cheletopsis* sp.	C 0, D 10, H 0 First record		First record
Erytraeidae			
*Abrolophus* spp.		Storage Dammam, Riyadh (Rostom 1993)	
Tetranychidae			
*Bryobia* sp.	C 1, D 0, H 6 First record	Tabuk, Riyadh, Qassim, Baha, Wadi e Hanifa (Alatawi and Kamran 2018)	First record
*Eutetranychus* sp.	C 4, D 0, H 12 First record	Throughout (Kamran et al. 2018)	First record
*Tetranychus* spp.	C 5, D 1, H 9 First record	Throughout (Alatawi and Kamran 2018)	Kuwait (Gamal-Eddin and Hamad 1992)
Mesostigmata			
Blattisociidae			
*Blattisocius keegani*		Storage Dammam (Rostom 1993), Jeddah; Riyadh (Rostom 1993)	
*B. tarsalis*		Storage Jeddah	
Laelapidae			
*Stratiolaelaps miles*	C 4, D 4, H 9 First record		Kuwait (Gamal-Eddin and Hamad 1992)
Macrochelidae			
*Macrocheles muscaedomesticae*	C 5, D 2, H 6 First record	Makkah Riyadh (Samšiňák 1979; Alatawi et al. 2018), Qassim (Fouly and Al-Rehiayani 2011)	First record
Macronyssidae			
*Ornithonyssus bacoti*	C Dammam (Al-Qurashi 2006)		
Melicharidae			
*Proctolaelaps pygmaeus*	C 5, D 3, H 11 First record	Makkah	First record
Oribatida			
Ceratozetidae			
*Ceratozetes* sp.	C 0, D 0, H 6 First record		First record
Oribatulidae			
*Oribatula tibialis*	C 16, D 2, H 28 First record	(Bayoumi and Al-Khalifa 1986)	First record
Scheloribatidae			
*Scheloribates* sp.	C 3, D 0, H 10 First record	(Bayoumi and Al-Khalifa 1986)	First record

^a^The table lists the mite species found in this study and any other species found in house dust in Saudi Arabia and Arabian Peninsula. C #, D #, H #: total number of mites collected from Coast, Desert, Highland samples in this study

### Astigmata, Sarcoptiformes, Acariformes

Excluding the results and analysis of *Dermatophagoides* spp, which are presented later.

*Aleuroglyphus ovatus* (Troupeau, 1879) (Acaridae, Acaroidea).

*A. ovatus* or the brown-legged grain mite (basionym *Tyroglyphus ovatus*).

There are some twenty reports of *A. ovatus* in house dust in Europe, Africa, and Asia (Somorin et al. 1978; Maurya et al. 1983; Suto et al. 1992; Yu et al. 2015). *A. ovatus* is present in house dust in Jeddah (Edrees 2008, 2009, 2010). Interestingly, the mite has not yet been collected from natural habitats in Saudi Arabia. The mite has also been retrieved from chicken houses in Israel (Mumcouglu and Lutsky 1990). In workers on poultry farms, the mite can enter the ear canal and cause tinnitus (Kato et al. 2011). It is a well-known stored product mite of some 35 commodities dominated by grains and flour but also found associated with food items like bread, candied fruit, bean curd, cheese, dried fish and shrimps, pasta, and processed food (Saleh et al. 1985; Hagstrum et al. 2013a). Patients allergic to *Dermatophagoides* species show crossreactivity to *A. ovatus* and *Chortoglyphus arcuatus* (Troupeau, 1879) (Puerta et al. 1993).

*Acarus siro* Linnaeus, 1758 (Acaridae, Acaroidea).

The grain mite *A. siro* (synonyms *Acaris farris* (Oudemans,* 1905)*,* Acarus farinae* Linnaeus, 1758, *Acarus farinae* De Geer, 1778, *Acarus farris* (Oudemans, 1905), *Aleurobius farinae* Canestrini, 1888, *Aleurobius farris* Oudemans, 1902, *Tyroglyphus farinae* (De Geer, 1778) has been detected in house dust in Jeddah (Edrees 2009, 2012). It is a well-known house dust mite with reports from many countries like Türkeyi (Aykut 2021), India (Gill and Dhaliwal 2018; Saw et al. 2024), Egypt (Heikal 2015a). It has been reported from litter in chicken houses in Israel some 35 years ago (Mumcouglu and Lutsky 1990), yet, it seems still to spread. The first confirmed sighting from Brazil, for example, is only from 2022 (Barbosa et al. 2022), however, sensitization has been detected earlier (Ferraroni et al. 2013). The species is known as a stored product mite, siro referring to a grain-storage pit, and Linnaeus finding it in flour, the species is now known from some 88 commodities including baby food, biscuits, cheese, bread, dried soup, and halva. The mite is deliberatedly added to the rind of the cheese mimolette to increase its flavour. In rare cases, it can cause oral mite anaphylaxis (Preiser-Funke and Bergmann 2023).

*Sancassania berlesei* (Michael, 1903) (Acaridae, Acaroidea).

This is a first record for the wet grain or mushroom musty mite, *S. berlesei* (basionym *Tyroglyphus berlesei*, synonyms *Caloglyphus berlesei* (Michael, 1903), *C. rodionovi* Zachvatkin, 1941, *Cologlyphus berleis lapsus duplex*), in house dust for Saudi Arabia and the Arabian Peninsula. It highly depends on wet environments, explaining why it was only found in Jeddah. It is a house dust mite recorded from many countries. It might contributes to atopy (Saxena et al. 1980). The species occurs in occupational ear acariasis of a worker in a waste processing plant in Spain (Poggioli and Taboada 2021) and caused tinnitis in a worker on a chicken farm in Japan. The mite is common in litter from chicken houses in Israel (Mumcouglu and Lutsky 1990). It is a stored product mite especially of some twenty commodities under slightly damp or mouldy conditions (Hagstrum et al. 2013a; EL-Sayed 2021). Egypt features several sister species as stored product mites, *S. betae* (Attiah, 1969), *S. krameri* (Berlese, 1881), *S. mycophagus* (Mégnin, 1874), and *S. rhicoglyphoides* (Zakhvatkin, 1937).

*Tyrophagus putrescentiae* (Schrank 1781) (Acaridae, Acaroidea).

The copra itch, ham or mould mite, *T. putrescentiae* (basionym *Acarus putrescentiae*), has been isolated from house dust in Jeddah (Edrees 2009, 2012). It feeds on moulds including entomopathogenic fungi (Ou et al. 2024). It is a formidable stored product mite affecting some 142 different commodities (Hagstrum et al. 2013a; EL-Sayed 2021; Klimov et al. 2024). It is also a threat to the poultry industry (Sulzbach et al. 2024). In Egypt, *T. zachvatkini* Volgin, 1948 serves as a stored product mite as well. There is cross-reactivity between the allergens of *Dermatophagoides farinae*, *D. pteronyssinus*, and *T. putrescentiae* (Chen et al. 2025).

*Carpoglyphus lactis* (Linnaeus, 1767) (Carpoglyphidae, Hemisarcoptoidea).

*C. lactis*, basonym *Acarus lactis*, has been collected from house dust in Jeddah (Edrees 2012). It is known as a house dust mite in Europe, North and South America, and Asia.

It is a storage mite too. Linneus described it as living in milk, and one of the German vernacular names is milk mite (Milchmilbe). However, it is better known as the dried fruit mite for its prevalence on sugar-rich substrates; some 40 commodities including wine corks carry this mite (Stejskal et al. 2015). Many beehives in Europe and the USA bear *C. lactis*, where they consume stored pollen; the mite also shows up in honey. In the production of sherry, some oak casks are colonised by such amounts of *C. lactis* that they lead to a higher floral and refreshing sensation, but also to a slightly increased bitterness in the final wine (Marín et al. 2009).

*C. lactis* and the acaroid species of concern here, *Aleuroglyphus ovatus*, *Acarus siro*, *Dermatophagoides farinae*, *D. pteronyssinus*, *Glycyphagus domesticus*,* Sancassania berlesei*, *Suidasia nesbitti*, and *Tyrophagus putrescentiae* can sometimes occur in such numbers in food products that they might cause urinary and intestinal acariasis; they have been recovered from human urine and feces (Li et al. 2003).

Some phytoseeid predatory species used in biocontrol are commercial reared on *C. lactis* as prey species (Cao et al. 2025).

The sister species, *C. ganzhouensis* Jiang, 1991 is found in house dust and on brown sugar in slaughter houses in China, while *C. munroi* (Hughes, 1952) in barn dust in Sweden, in bee hives in Czechia, and on spider webs in the UK.

*Blomia freemani* Hughes, 1948 (Echimyopodidae, Glycyphagoidea).

Edrees (2009); (Edrees 2012) found *B. freemani* in dust of houses, motels, and hotels in Jeddah. In Alexandria, Egypt, *B. freemani* is one of the dominant species in house dust (Rezk et al. 1996). Although originally described from the Northern Ireland, reports from house dust in Europe (Switzerland, Wales) and South America (Colombia) are rare, while it is quite common in house dust in China and India. In China, *B. freemani* together with *Dermatophagoides farinae* and *D. pteronyssinus* were more abundant in mattress dust than in house dust (Lai 1988). The breeding rate of acaroid mites like *B. freemani* was the highest in taxis, followed by private cars, but less so in buses (Wang and Li 2012).

Like several other mite species contaminating stored flour, *B. freemani* has caused oral mite anaphylaxis (pancake syndrome) (Wen et al. 2005). *B. freeman* has originally been described as a mite of stored wheat, it is a storage mite of rice, millet, sweet potato chips, and Chinese medicine.

In addition to *B. tropicalis*, sister species *B. kulagine* Zakhvatkin 1942, *B. thori* Zakhvatkin 1942, and *B. tjibodas* (Oudemans, 1910) are also associated with house dust.

*Blomia tropicalis* Van Bronswijk, De Cock and Oshima 1973 (Echimyopodidae, Glycyphagoidea).

*B. tropicalis* has been repeatedly described from house dust in Jeddah (Edrees 2008, 2009, 2010, 2012). It sometimes occurs together with *B. freemani* (Edrees 2009, 2012). It has a rich medical history (Caraballo et al. 2024). Severe cases of allergic rhinitis can result from dual sensitization to *D. pteronyssinus* and *B. tropicalis* (González-Pérez et al. 2020). Patients with allergic rhinitis sensitized to *B. tropicalis* might be most vulnerable to air pollutants (Quek et al. 2019). There are several vaccine candidates based on *B. tropicalis* (Castro-Almarales et al. 2020; da Silva et al. 2020). It is also a stored product mite of rice and tabacco.

*Glycyphagus domesticus* (de Geer, 1778) (Glycyphagidae, Glycyphagoidea).

This is a first record for *Glycyphagus* and *G. domesticus* (basionym *Acarus domesticus*) in house dust for Saudi Arabia and the Arabian Peninsula. As the species epithet suggests, *G. domesticus* is known as the house mite. Already in 1983, *G. domesticus* has been recovered from soil in the Riyadh region (Al-Khalifa and Bayoumi 1983). *G. domesticus* in house dust, for example, in Egypt has been published twice (Heikal 2015a, b), in Türkiye (Mutlu et al. 2019), France (Reboux et al. 2019), or Poland (Solarz et al. 2021). It can build massive colonies on furniture stuffed with not thoroughly cleaned horse hair (Oudemans 1897). It feeds on the fat still adherend to the hair. Together with *Acarus siro* and *Lepidoglyphus destructor*,* G. domesticus* can cause oral mite anaphylaxis (Mulhall and Conlon 2019). It is a stored product mite of some 44 c ommodities. In addition, in Egypt, *G. aegyptiacus* Attiah, 1971, and *G. oryzae* Attiah, 1971 are known as stored product mites.

*Histiostoma feroniarum* (Dufour, 1839) (Histiostomatidae, Histiostomatoidea).

This is a first record for *Histiostoma* and *H. feroniarum* (basionym *Hypopus feroniarum*), synonyms *Tyroglyphus rostroserratus*, *Acarus mamillaris*, in house dust for Saudi Arabia and the Arabian Peninsula. There might be three specimens of *H. feroniarum* collected in Saudi Arabia in the Royal Belgian Institute of Natural Sciences, Brussels, Belgium according to the Global Biodiversity Information Facility. They are not obvious in the museums catalogue. The only species published from Saudi Arabia is *H. sammari* Eraky & Shoker, 1994 phoretic on ant species (Mashaly et al. 2011). *H. feroniarum* is known from house dust in several countries, for example, China (Li et al. 2005), island of Tenerife, Spain (Sanchez-Covisa et al. 1999), Venezuela (Hurtado and Parini 1987) or Australia (Colloff et al. 1991). *H. humidiatus* (Vitzthum, 1927) and *H. sapromyzarum* (Dufour, 1839) are fellow house dust mites (Lee and Cho 1984; Colloff et al. 1991). *H. feroniarum* is a stored product mite of bread, wet grain, dried fruit, dried vegetables, and Chinese medicine. *H. feroniarum* is common in mushroom houses (Szafranek and Lewandowski 2017; Qu et al. 2023). *H. onioni* Eraky & Shoker, 1994 and *H. sarrai* are stored product mites of onions in Egypt.

*Suidasia pontifica* Oudemans, 1905 (Suidasiidae, Acaroidea).

This is a first record for *S. pontifica*, the scaly grain mite, in house dust for Saudi Arabia and the Arabian Peninsula. There is some confusion about the naming of the species. In 1978, *S. medanensis* Oudemans 1924 was synonymized with *S. pontifica* Oudemans 1906, retaining the original and therefore the correct name, *S. pontifica* (Fain and Philips 1978). This was never opposed; yet, *S. medanensis* is now more often used in the understanding that they are the same species. *S. melanensis* in the literature is a spelling error. *S. pontifica* is widespread as a house dust mite (Somorin et al. 1978; Colloff et al. 1991; Mariana et al. 2000; Modak et al. 2004). Some commercial skin prick test solutions in India for *Dermatophagoides pteronyssinus* or *Blomia tropicalis* contained instead proteins of *S. pontifica* (Huber et al. 2021). The importance of *S. pontifica* as a stored product mite is even greater. Together with its sister species, *S. nesbittii* Hughes, 1948, it is found in some 55 commodities (Hagstrum et al. 2013a; Bakr et al. 2022; Ta-Phaisach et al. 2023). It is associated with a variety of food products. The large occurrence in a pancake mix in Panama lead to the pancake syndrome in the form of oral mite anaphylaxis (Barrera et al. 2015; Sánchez-Borges et al. 2020). The pancake syndrome can also be induced by other mite species like *Dermatophagoides farinae* (Erben et al. 1993; Kano and Murata 2025), *D. pteronyssinus*, *Tyrophagus putrescentiae* (Takahashi et al. 2014), *Acarus siro* (Preiser-Funke and Bergmann 2023), and the booklouse *Liposcelis bostrychophila* Badonnel, 1931 (Matsumoto et al. 2025). Oral mite anaphylaxis might also look like an acute asthma attack (Garcia et al. 2016). *S. pontifica* was found together with *Tyrophagus putrescentiae*, *Blattisocius tarsalis* (Berlese, 1918), and *Blomia tropicalis* in peanut bars and milk sweets in Brazil (Franzolin et al. 1994).

*Suidasia nesbitti* Hughes, 1948 (Suidasiidae, Acaroidea).

*S. nesbitti*, the scaly grain or wheat pollard itch mite, has been found in house dust of Jeddah several times (Edrees 2008, 2009, 2012). It is reported from Europe, the Middle East, and Asia as a dust mite. It is also a cosmopolitan pest of grains and feed high in protein and fat (Devi et al. 2022). *S. nesbiiti* has been collected from as a soil mite from soil of a moss-turf area and the wet soil from a kitchen liquid waste collection area of the Schirmacher Oasis in East Antarctica (Sanyal 2004).

### Prostigmata, Trombidiformes, Acariformes

*Cheyletus eruditus* (Schrank 1781) (Cheyletidae, Cheyletoidea).

This is a first record for *C. eruditus* (basionym *Acarus eruditus*, syn. *C. butleri* Hughes, 1948, *C. desitus* Qayyum & Chaudhri, 1977, *C. ferox* Trouessart, 1889, *Cheyletus ferox* Banks, 1906, *C. seminivorus* Packard, 1878, and others) in house dust for Saudi Arabia and the Arabian Peninsula. It is also known under vernacular names like grain itch mite, mattress itch mite, prairie itch mite, and so on, however, Schrank named it a Büchermilbe, book mite, when he described it from the bookbinder’s glue of humid books, making it a true domestic mite (Schrank 1781). This predator mite has been associated with maize grains and rodents in Saudi Arabia (Al-Youssif and Soliman 1979; El-Bahrawy and Al-Dakhil 1993; Alatawi and Kamran Ahmad 2017). *C. eruditus* or its sister species, *C. malaccensis* (Oudemans, 1903) are almost always found in house dust samples (Liu et al. 2010; Saw et al. 2018; Zhou et al. 2022; Karuthedath and Raghavan 2024). *C. malaccensis* has been found in house dust in Jeddah city (Edrees 2008). The two species are the main predators of the Dermatophagoides species in house dust. Although Dermatophagoides species have been extensively associated with atopic diseases, *C. eruditus* can also be of clinical relevance for persistent, non-occupational allergic rhinitis and skin symptoms (Poza Guedes et al. 2016; Hsu et al. 2024; Castromil-Benito et al. 2025). As a stored product mite, it is also associated with more than 53 commodities ranging from grains, spices, seeds, dried fruits to Chinese medicine (Hagstrum et al. 2013a). The species is commercially used as a biocontrol agent against a number of grain mites, in particular *Acarus siro* and *Lepidoglyphus destructor* (Schrank 1781) in agricultural warehouses.

*Cheyletus malaccensis* (Oudemans, 1903) (Cheyletidae, Cheyletoidea).

*C. malaccensis*, basonym *Cheletes malaccensis*, synonyms Cheletes fortis Oudemans, 1904, *C. vorax* Oudemans, 1903; *Cheyletus avidus* Qayyum & Chaudhri, 1977, *C. ayyazi* Akbar, Aheer & Ishtiaq, 1993, *C. baridos* Akbar, Rahi & Chaudhri, 1988, *C. caucasicus* Zachvatkin, 1949, *C. egypticus* Elbadry, 1969, and some ten more.

What applies to *C. ereditus* also applies to *C. malaccensis*. As a predator species it is found in house dust as well as in stored products. Although it does not carry a commonly used vernacular name, it has been encountered in more countries with a truly worldwide distribution, and it affects even a wider diversity of commodities than *C. ereditus*. As a biocontrol agent, it is newer. *C. malaccensis* prefers a slightly warmer climate; *malaccensis* refers to Malacca (Melaka), Malaysia. It predates better on booklice (Hu et al. 2025) and it is considered for biocontrol in poultry farms. The expression of heterotrophic males is much more pronounced in *C. malaccensis*. Both species engage in cannibalism if prey is scarce. The colour of *C. malaccensis* changes with the prey it consumes, yellowish with *Aleuroglyphus ovatus* and *Carpoglyphus lactis*, blackish with *Lasioderma serricorne* Fabricius, 1792, and brown spots with *Cryptolestes pusillus* (Schönherr, 1817) (Han et al. 2025). When *C. malaccensis* bites, meaning stabbing human skin, it tries to feed and causes papules (Yoshikawa 1987).

*Cheletopsis* sp. Oudemans, 1904 (Cheyletidae, Cheyletoidea).

This is a first record for *Cheletopsis* in house dust worldwide. It is also a first record for this genus for Saudi Arabia, the Arabian Peninsula, and Asia. *Cheletopsis* species are mostly known from feather quills and bird’s nests (Bochkov et al. 2002; Bochkov and Skoracki 2012). They predate on other mite species.

*Tetranychus* spp. Dufour, 1832 (Tetranychidae, Tetranychoidea).

This is a first record for *Tetranychus* in house dust for Saudi Arabia. The red spider mites, *T. evansi* Baker & Pritchard, 1960, *T. neocaledonicus* André, 1933, *T. salicornicus* Alatawi and Kamran 2018; T. *turkestani* (Ugarov & Nikolskii, 1937), and *T. urticae* Koch, 1836 (Alatawi and Kamran 2018) are known from Saudi Arabia; *T. salicornicus* is a new species described from Riyadh. Tetranychidae have been reported early on as part of the house dust fauna (Solomon 1961), and they are often mentioned in the house dust literature but rarely identified to the genus or species level. *Tetranychus* species occur in houses worldwide, for example, in Australia, Colombia, Iceland, Türkiye (Colloff et al. 1991; Hallas et al. 2004; Navarro et al. 2008; Zeytun et al. 2015, 2016) and Kuwait (Gamal-Eddin and Hamad 1992).

*Eutetranychus* sp. Banks, 1917 (Tetranychidae, Tetranychoidea).

This is a first record for the genus *Eutetranychus* in house dust for Saudi Arabia and the Arabian Peninsula. *E. africanus* (Tucker, 1926), *E. banksi* (McGregor, 1914), the citrus brown mite, *E. orientalis* (Klein, 1936), *and E. palmatus* Attiah, 1967 are known from Saudi Arabia (Kamran et al. 2018). *E. orientalis* has been found in house dust in India (Modak et al. 2004).

*Bryobia* sp. C.L.Koch, 1835 (Tetranychidae, Tetranychoidea).

This is a first record for *Bryobia* in house dust for Saudi Arabia and the Arabian Peninsula. Four species are known from Saudi Arabia, *B. alveolata* Auger & Flechtmann, 2009 originating from Tunesia, the clover mite, *B. praetiosa* Koch, 1836, *B. rubrioculus* (Scheuten, 1857), and *B. tuttlei* Smiley & Baker, 1995 originating from Yemen (Alatawi and Kamran 2018). There are some 149 named species in this genus (Mirza et al. 2025). *B. praetiosa* is known from house dust in Italy and Israel (Castagnoli et al. 1983; Mumcuoglu and Shalom 2013). While numbers of *Bryobia* mites in house dust are normally in the single digits, mainly in the past in Europe and North America, often new houses had been invaded in the spring by hundreds of thousands, of grass mites, *B. praetiosa* or *B. gramium* (Schrank 1781) (syn. *B. cristata* (Dugès, 1834), leading, for example, to a quarter of a million on the floor of a bedroom in the USA (English and Snetsinger 1957; Anderson and Morgan 1958; Rack 1984; Mumcuoglu and Shalom 2013). In England, new police headquarters and Magistrates’ courts were overrun by such high numbers of mites that they had to be closed (Parkin 1958). The buildup of large populations in just one or two years is facilitated by parthenogenetic reproduction in many species. *B. gramium* has been described from house dust of rural farms in Iceland (Gígja 1944; Guðmundsson et al. 2008). Unidentified species of *Bryobia* have been collected from house dust in Czechia, in the living and bed rooms of houses in Türkiye, mattresses in Denmark, and in a house in Israel (Costa 1978; Vobrázková et al. 1979; Andersen 1984; Zeytun et al. 2016). These mites often develop in sunny places where grass reaches directly the wall of houses and buildings, which was often the case in new urban or suburban developments of the 1950’s to the 1980’s. The mites are very mobile and enter the houses mostly through open windows by accident in search for food, mainly grass, which they will not find indoors; eventually starving to death not withstanding any house plants. Large amounts of eggs might be deposited on outside window frames. The mites climb up the walls of buildings up to the fourth floor. When crushed, they leave green stains behind. They do not attack humans or animals.

### Mesostigmata, Parasitiformes

*Proctolaelaps pygmaeus* (Müller, 1859) (Melicharidae, Ascoidea).

This is a first record for *P. pygmaeus* (basionym *Gamasus pygmaeus*, and some 20 synonyms including *Lasioseius ventritrichosus* Schweizer, 1949, *Garmania alpina* (Schweizer, 1949) in house dust worldwide. It has been found in wheat floor in Jeddah and in manure of chicken farms in the Makkah region. At other places, it is associated with mouldy barley, bulbs, and timber, hay, rice and other grains. The mite feeds on fungi and predates on small arthropods. It stands out for being able to feed on solid food. It can bite humans and cause extensive papular dermatosis; this has been reported from timber workers in Australia and New Zealand (Halliday et al. 1998). Damp construction wood possibly introduced it into a newly constructed office building in Berlin, Germany.

*Macrocheles muscaedomesticae* (Scopoli, 1772) (Macrochelidae, Eviphidoidea).

Surprisingly, this is a first record for *M. muscaedomesticae* (basionym *Acarus muscae domesticae*), synonym *Macrocheles lundae* Krantz, 1970, for house dust worldwide. Wherever there is the house fly *Musca domestica* Linnaeus, 1758, vinegar flies like *Drosophila* Fallen, 1823 species, stable flies *such as Stomoxys calcitrans* (Linnaeus, 1758) or scuttle flies like *Megaselia* Rondani, 1856 species, one might expect to find *M. muscaedomesticae* as well (Perotti 2001; Perotti and Braig 2009). The manure of cattle sheep and poultry farms in the Makkah region all contained this species It has been associated with dried fish, grain, maize trash, onion bulbs, mouldy peanuts, and rotting wheat residues elsewhere. *M. muscaedomesticae* have also been associated with human corpses (Braig and Perotti 2009; Che Kamaruzaman et al. 2018).

*Ornithonyssus bacoti* (Hirst, 1913) (Macronyssidae, Dermanyssoidea).

*O. bacoti*, basonym *Leiognathus bacoti*, has a long list of synonyms including *Bdellonyssus nagayoi* (Yamada, 1930), *Hirstionyssus musculi* Domrow, 1963, *Liponissus lutzi* Fonseca, 1942, *Liponyssus meprai* Manso Soto & Pletneff, 1951, *Macronyssus bacoti* (Hirst, 1913). It is known as the tropical rat mite. The adjective tropical is slightly misleading as it now can be found worldwide. It is a blood-feeding mite that causes a non-specific dermatitis in humans and is often un- or misdiagnosed. Bites might occur in a cluster. The mite leaves its host after bloodfeeding. Many pathogens and parasites have been transmitted in laboratory experiments, but its role as a vector to humans is still doubted with the exception of the bacterium *Bartonella henselae* causing cat-scratch disease (Bradley et al. 2014). Unfed protonymphs have survived up to 43 days (Sudd 1952), Kelaher et al. (2005) claim survival of the mite for up to 63 days without a bloodmeal. *O. bacoti* is able move over longer distances, which means it can be found in houses and workplaces that are not infested by rodents (Theis et al. 1981). This implies that an infestation with this mite is not necessarily associated with run-down homes or workplaces but can occur also on modern, well-maintend premises (Kelaher et al. 2005).

The mite shows little host specificity, even humans can act as an alternative host. It can associate with a wide diversity of pet, companion, and domesticated animals, examples are African pygmy hedgehog in Germany (van Well and Beck 2022), donkey in France (Dumitrache et al. 2023), hamsters, gerbils, guinea pigs, rabbits, degus, kangaroo mice, hedgehogs, tricolour squirrels, and sugar gliders in Italy (d’Ovidio et al. 2018), dogs in the USA (Bradley et al. 2014), in Japan (Ogata et al. 1978), cats (Baker 1999), and chicken (Ponnudurai et al. 2015; Villanueva et al. 2020). The owners of the animals might become infested as well.

On this description, one would not expect finding this mite in house dust or in large numbers. However, in the port city of Dammam in Saudi Arabia, from the dust of houses of children suffering allergenic manifestations, *D. farinae* and *O. bacoti* were recovered (Al-Qurashi 2006). Although *O. bacoti* has not been reported from house dust for any other country of the Arabian Penninsula, the mite has been found on rats in Qatar (Islam et al. 2023) and should be assumed to be present over the entire Arabian Penninsula. Outside Saudi Arabia, dermatitis patients in Egypt had *D. pteronyssinus*, *O. bacoti* and *Haemogamasus pontiger* (Berlese, 1904) in their house dust (Morsy et al. 1994). In rural houses in Egypt, *O. bacoti* was the most numerous mite in house dust, more abundant even than pyroglyphid species (El-Shazly et al. 2006). In Japan, the density of *O. bacoti* is between 16 and 530 mites per gram of house dust, with little difference between houses built from wood or concrete (Toma et al. 1993b). Infestations have been reported from older student accommodation in Edinburgh, Scotland (Sargison et al. 2025). In ten kindergardens in Iran, 20% (*N* = 342) of the mites collected from dust were *O. bacoti*, only 18% *D. farinae* (Soleimani-Ahmadi et al. 2017).

*Stratiolaelaps miles* (Berlese, 1892) (Laelapididae, Dermanyssoidea).

This is a first record for *S. miles* (basionym *Laelaps miles*, syn. *Hypoaspis miles* (Berlese, 1892) for Saudi Arabia. It has been reported from house dust in Kuwait and India (Gamal-Eddin and Hamad 1992; Modak et al. 2004). It is a stored product mite of decaying oatmeal spillage and wheat residue. The mite predates on flies, thrips, springtails, and other mites. Owners of pet reptiles, amphibians and invertebrates like spiders use this species to prevent or treat infestations by other mites. In the snail breeding industry, it is used against the parasitic while snail mite *Riccardoella limacum* (Schrank, 1776) (Ereynetidae). Mushroom farms use it against the *Lycoriella* Frey, 1942 sciarid fly and fungus gnats. The widespread use as a biocontrol agent has led to IgE sensitization against this species in greenhouse workers (Kronqvist et al., 2005). There has been some confusion in the biocontrol industry about the identity of *S miles* and *S. scimitus* (Womersley, 1956) (Walter and Campbell 2003).

### Oribatida (Sarcoptiformes, Acariformes)

*Oribatula tibialis* (Nicolet, 1855) (Oribatulidae, Oripodoidea).

This is a first record for *O. tibialis* (basionym *Notaspis tibialis*, and some 16 synonyms) in house dust worldwide. O. *tibialis* has been recorded from sandy soil under ficus trees in Samitah, Saudi Arabia (Bayoumi and Al-Khalifa 1986). There is also one report from Oman (Vasar et al. 2022). The high abundance soil mites (Oribatida) of the family Oribatulidae seems unusual for indoors. However, Oribatida highest prevalence in the highland region matches with their natural habitat, areas with tree cover, more vegetation that can sustain Oribatida communities (Galal et al. 2021). In fact, the Oribatida is a poorly known group of mites from the Arabian Peninsula. Aside from being a soil mite it is also known as a ‘storaged timber mite’. It can be found in wood chips (Klimek and Chachaj 2018). It has been implicated in dermatitis found on the trunk and arms of wood workers (Liguori et al. 1989). The species stands out as one of a few animals releasing hydrogen cyanate as a chemical defence against predators (Brückner et al. 2017). Lichens and bird nests are additional habitats (Melekhina 2023; Melekhina et al. 2023). Because of its small size, a subspecies is living amidst the adhesive traps on leaves of a carnivorous plant (Antor and Garcia 1995). *O. tibialis* is becoming common in European cities (Mangová et al. 2019). What we see here in Saudi Arabia is the transition of this species to a house dust mite. *O. tibialis* is a stored product mite of dried apricots in Türkiye (Çobanoğlu 2008). One *Oribatula* species has been reported from house dust in Korea and China, *O. sakamorii*, now *Phauloppia sakamorii* (Aoki, 1970) (Ree et al. 1997; Chen et al. 2023). Unidentified species of Oribatulidae have been seen in house dust of Japan and Colombia (Oshima 1970; Charlet et al. 1977; Suto et al. 1991; Hatsushika and Miyoshi 1992; Toma et al. 1993a).

*Scheloribates* Berlese, 1908 sp. (Scheloribatidae, Oripodoidea)

This is a first record for *Scheloribates* in house dust for Saudi Arabia and the Arabian Peninsula. *Andeszetes* Hammer, 1961, *Megascheloribates* Lee & Pajak, 1990, *Neoscheloribates* Hammer, 1973, *Paraschelobates* Jacot, 1935, *Protoschelobates* Jacot, 1934, *Semischeloribates* Hammer, 1973, and *Storkania* Jacot, 1929 are synonyms of the genus *Scheloribates.* Four to six species of *Scheloribates* (Bayoumi and Al-Khalifa 1986; Kardar 1988) are known form soil and leaf litter in various places of Saudi Arabia: *S. fimbriatus* subsp. *africanus* Wallwork, 1964 (AI-Khalil, Khamasin), *S. laevigatus* (Koch, 1835) (Riyadh, Durma, Jalajil, Al-Zulfi, Al-Qatif, Al-Sayl Al-Kabir, Samitah), *S. pallidulus* (Koch, 1841) (Al-‘Uwaynah, Al-Surrah), *S. riyadhiensis* Kardar 1988 (Riyadh), now considered a *species inquirenda* by Subías (2022), *S. saudicus* Bayoumi & Al-Khalifa, 1985 (l-Kharj, l-Dir’iyah) and *S. saudiensis* Kardar 1988 (Riyadh), now also considered a *species inquirenda* by Subías (2022). *S. sacculipunctatu*s Mahunka 2009 is a new species from leaf litter near Al-Hayer in the UAE (Kardar 1988; Mahunka 2009). Species of *Scheloribates* have been found in the dust of several houses in Brazil (Rosa and Flechtmann 1979) and Colombia (Charlet et al. 1977), S. *laevigatus* has been found in house dust in China (Chen et al. 2023), S. *latipes* (Koch, 1844) now a subspecies of *S. pallidulus*, the same species that is the most common *Scheloribates* in Saudi Arabia, has been found in house dust in Korea (Ree et al. 1997) and *rufafulvus*, now *Protoribates (P.) rufafulvus* (Kardar, 1977) and considered a *species inquirenda* by Subías (2022); and *S. translamellaris* (Kadar, 1977), now *S*. *praeincisus* subsp. *sandvicensis (Jacot*,* 1934)*, in house dust in India (Maurya and Jamil 1982). *S. laevigatus* is also a pest of stored barley, rice, and wheat grains and mouldy grains, potatoes and floor debris (dust) in Iran, Japan, USA, and Europe (Ostovan 1993; Hagstrum et al. 2013a).

*Ceratozetes* Berlese, 1908 sp. (Ceratozetidae, Ceratozetoidea)

This is a first record for *Ceratozetes* in house dust worldwide. It is also a first record for this genus and family for Saudi Arabia and the Arabian Peninsula. The mites inhabit soil, litter, and canopy habitats. Many are adapted to colder and montane areas. There are currently some 48 species in the genus *Ceratozetes* (Behan-Pelletier and Eamer 2009; Subías 2022). Species geographically closest to the Arabian Peninsula are *C. baleensis* Ermilov et al. 2011 d *gracilis* (Michael, 1884) in forest soil of mountains in Ethiopia (Ermilov et al. 2011)d *conjunctus* Mihelcic, 1956 and *C. macromediocris* Shaldybina, 1970 in Iran (Akrami 2015). *C. conjunctus* has also been found in soil of industrial sites of the Basque Country (Corral-Hernández and Iturrondobeitia 2012). *Ceratozetes* has not been associated with any stored products.

### Analysis of mite families and association with main predators

**Fig. 2:**
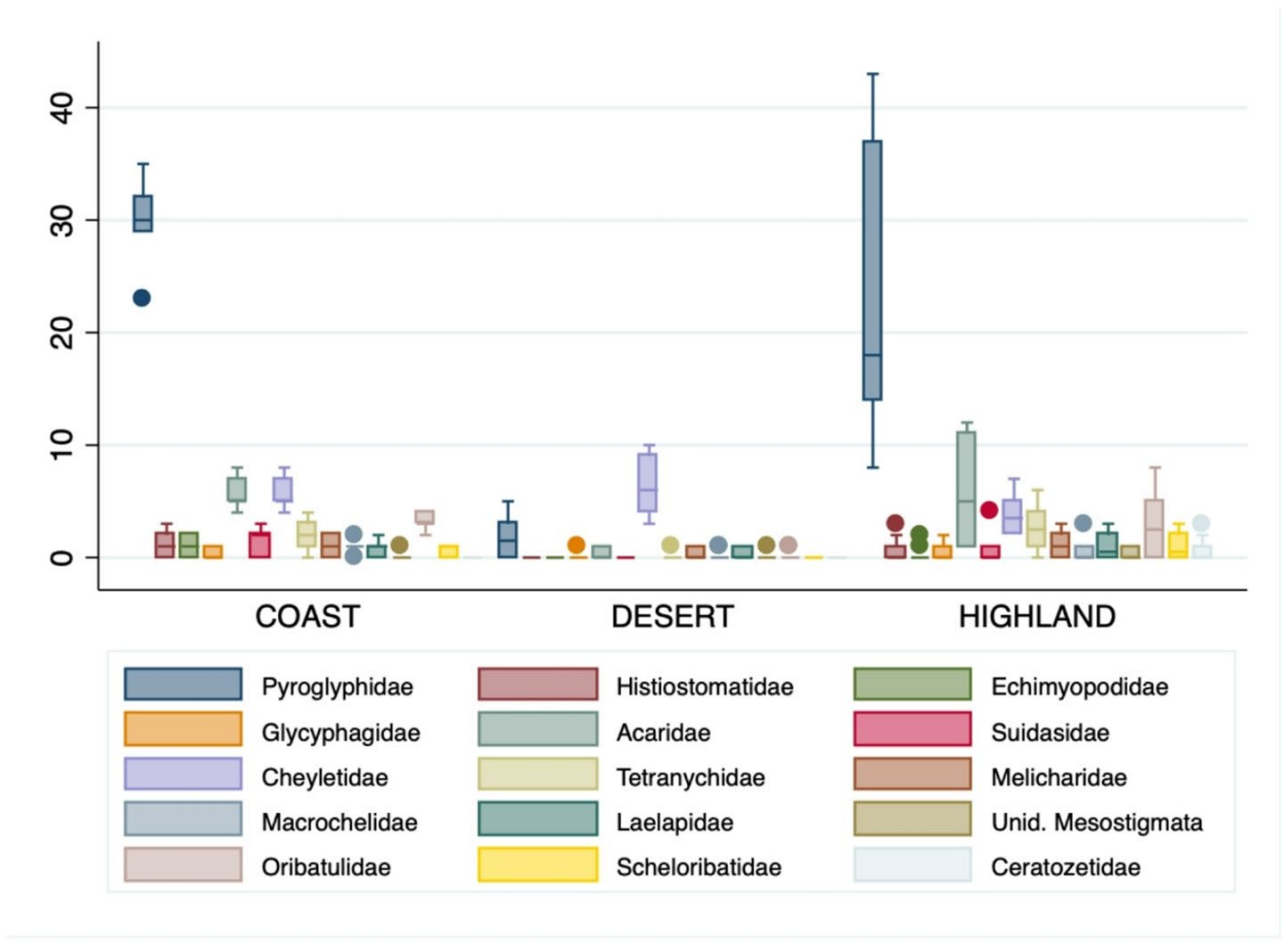
Boxplots of counts of mites within families by region. Boxplot description: Median, central line; box, lower and upper quartiles; external lines, lower and upper extremes; dots, outliers.

Our study on all families of house dust mites found in Saudi Arabia, which corresponds to the 2nd sampling attempt (2018), shows a diverse mite composition and variation between regions (Fig. 2; Table S1). From a general view, the most abundant mites belong to the families Pyroglyphidae (N_total_ = 400), followed by mites from the families Cheyletidae (N_total_ = 127) and Acaridae (N_total_ = 115). Oribatid soil mites (N_total_ = 65) were unexpectedly numerous in the house-dust (families Oribatulidae, Ceratozetidae and Scheloribatidae). The remaining families did not reach a 50 counts threshold for their inclusion in the analysis.

Considering the total of HDM found, their abundance significantly differs in numbers between the three main regions (X^2^: 16.302, df: 2, *P* < 0.001). In particular while Pyroglyphidae had borderline variation in abundance (significance) between the three climatic regions (X^2^: 5.692, df: 2, *P* = 0.0551), their main predator species, *C. eruditus* showed consistent differences comparing regions (X^2^: 17.579, df: 2, *P* < 0.001). When compensating for data dependency, by pairing Pyroglyphidae with *C. eruditus*, the two populations varied significantly in each region (by equal means, *P* < 0.001 for all regions), and *C. eruditus* had a significant effect on populations of *D. farinae* in the desert region (GLM, *P* < 0.01). Using families Pyroglyphidae and Acaridae as independent variables, there was interaction between *C. eruditus* and Pyroglyphidae (GLM, *P* < 0.01) in the desert, while no interaction with Acaridae was observed in any region.

## The Indicator Species Analysis (IndVal)

The bird/mammal skin cheyletid, *Cheletopsis* sp. resulted as a significant marker for the desert. *D. pteronyssinus*, *Histiostoma* sp. and *Ceratozetus* sp. were found significantly associated with the highlands, while *Dermatophagoides* n. sp. resulted in the highest, significant association with the coastal region, followed by *Aleuroglyphus* sp. and *Histiostoma feroniarum*.

### Detailed analysis of *Dermatophagoides* species

**Fig. 3:**
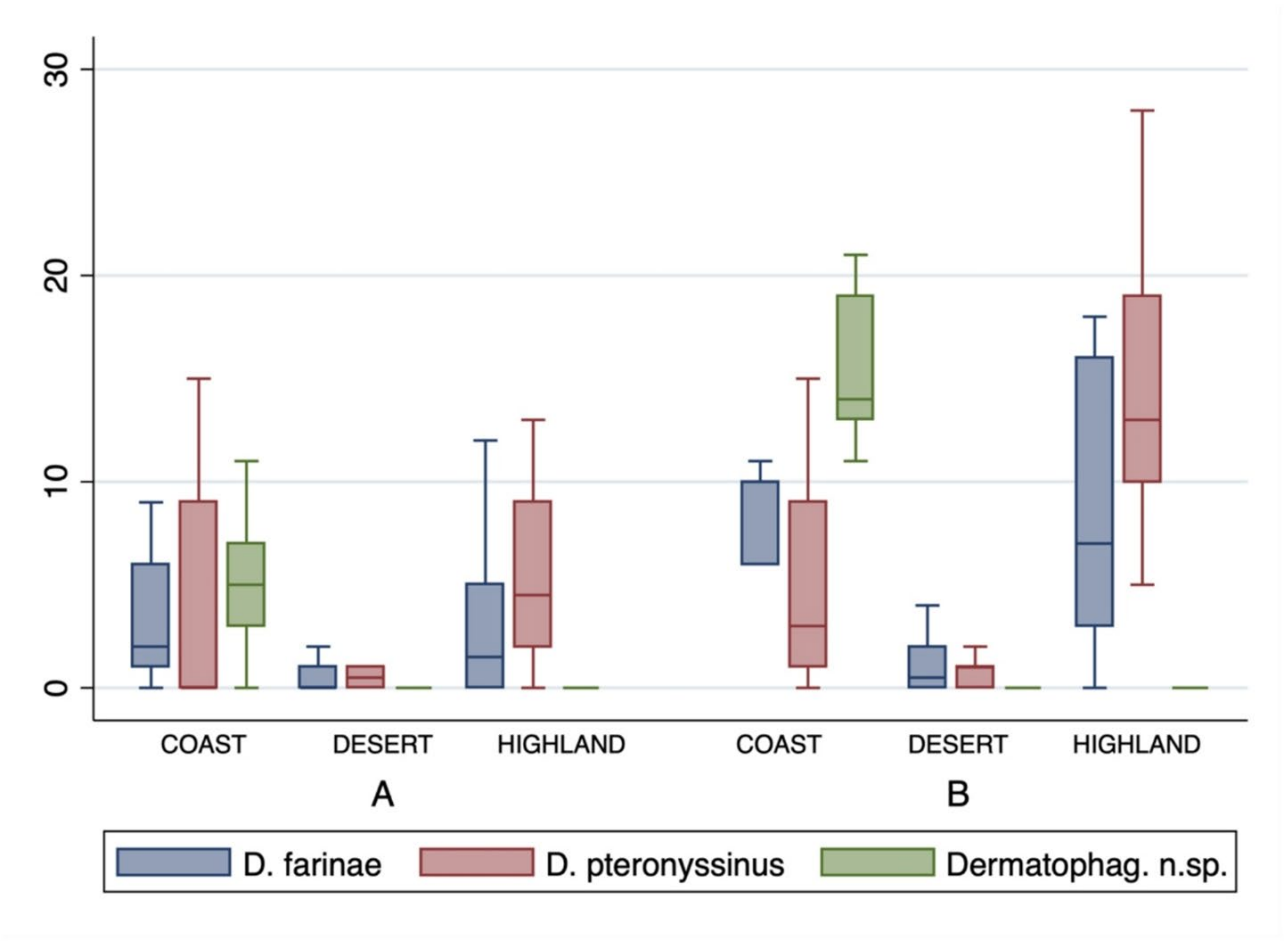
Boxplots of counts of *Dermatophagoides* mites by species, *D. pteronyssinus*, *D. farinae* and *Dermatophagoides* sp. n. (new species), by region and sampling (A; 1 st sampling and B: 2nd or repeated sampling). Boxplot description: median, central line; box, lower and upper quartiles; external lines, lower and upper extremes; dots, outliers

From the data of the repeated sampling, 2 × 25 samples a total of 566 specimens within 3 species were collected from the 3 regions: *Dermatophagoides pteronyssinus* (N_total_ = 262), *D. farinae* (N_total_ = 200) and *Dermatophagoides* n. sp. (N_total_ = 104) (Fig. 3, Table S2). For none of the three species the data follow a linear distribution, even by pooling numbers of mites in regions (Shapiro-Wilk W test, *P* < 0.0001, in all 3 cases). Between regions, pairwise correlations were different between *D. farinae* and *Dermatophagoides* n. sp. in the coast (*P* = 0.05, borderline), and *D. farinae* and *D. pteronyssinus* in the highlands area (*P* < 0.001).

Kendall correlation coefficient suggests a positive correlation (concordant ranking) between *D. farinae* and *D. pteronyssinus* (*P* < 0.01) regardless of region, while *Dermatophagoides* n. sp. shows low or no correlation (is discordant) with the other two species, suggesting an independent distribution pattern. Within regions, there was a highly positive correlation between *D. farinae* and *D. pteronyssinus* in the highlands (*P* < 0.01). In other words, there is an association between these species. They occur together in the dust samples collected from different Saudi Arabian households.

Our studies of species interaction showed that within Saudi Arabian houses the common predator of Pyroglyphidae, *C. eruditus*, follows the same regional distribution pattern of Pyroglyphidae, specifically targeting populations of *D. farinae* in the desert. Interestingly, Saudi populations of *C. eruditus* seem not to significantly affect Acaridae species; but this could be due to the smaller samples of Acaridae in Saudi houses, compared to other regions of the world (Smiley 1991; Bertone et al. 2016). Predatory cheyletids are common in house dust, preying on other micro-arthropods (such as pyroglyphid mites), moving indoor via phoresy and/or entering associated with pets, as well as on domestic plants or on the humans themselves (Colloff 1998, 2009). Cheyletidae were abundantly found in the house-dust from the desert area, thus may be associated with a dry hot climate that might only sustain Pyroglyphidae due to the humidity produced by air conditioning (Van Strien et al. 2004; Thomas 2010). Acaridae, on the other hand, are quite prevalent in temperate regions of the world. Acaridae species have lower temperature thresholds.

Pyroglyphidae, Acaridae, and Oribatulidae are positively correlated. The three groups can utilise same food resources such as fungi and detritus, given the right environmental conditions, especially humidity (Arlian 1992; Arlian and Dippold 1996; Arlian and Morgan 2003; Colloff 2009; Klien and Walzl 2010). For Pyroglyphidae, this is perhaps due to the frequent humidity bursts combined with the right temperatures in these climatic regions (Hughes 1976; Zhang 1998; Colloff 2009; Thomas 2010); they favour indoor environments with high levels of humidity (Klien and Walzl 2010). This correlation can also be linked to the confirmed (negative) interaction with their main predator, *C. eruditus*.

The other unidentified Cheyletidae species found in this study, *Cheletopsis* sp., a bird mite, was only recorded from desert samples. Its occurrence in the desert helps explain that desert habitation is perhaps more exposed to the visit of wild birds and/or mammals (Hughes 1976; Bochkov 2002; Walter et al. 2009).

**Fig. 4:**
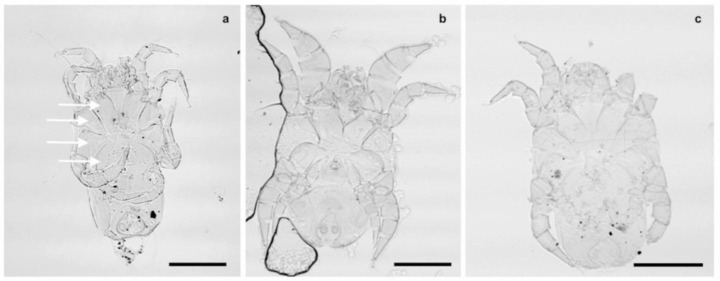
*Dermatophagoides* mite species from Saudi Arabia, ventral view of males of (a) *D. pteronyssinus*, (b) *D. farinae* and (c) *Dermatophagoides* n. sp. . Bar, 100 μm, arrows showing epimeral plates

Of particular interest is the occurrence of a third, new species of *Dermatophagoides*, restricted only to the coastal region (*Dermatophagoides* n. sp.). The differences of the three *Dermatophagoides* species are shown in Fig. 4. This new species, which is currently being described, plus the prevalent *Aleuroglyphus* and *Histiostoma feroniarum* could become reliable biomarkers for location, as traces of potential utility in forensic investigations (Hani et al. 2018).

## Conclusions

Dust mites in Saudi Arabia are much more divers than expected with first records, species for the first time associated with house dust, and mite species likely new to science. This work shows that some dust mite species are specific for climate regions. It differentiates the importance of HDMs for allergies in coastal, desert, and highland areas; and it reviews all known HDMs for Saudi Arabia.

## Supplementary Information

Below is the link to the electronic supplementary material.

Supplementary file1 (XLSX 12 KB)

Supplementary file1 (XLSX 12 KB)

## Data Availability

All data generated or analysed during this study are included in this published article.
