# Correction: House dust mites in three contrasting climatic regions of Saudi Arabia

**DOI:** 10.1007/s10493-026-01129-8

**Published:** 2026-04-08

**Authors:** Riyadh Hussain S. Aeban, Medjedline Hani, Henk R. Braig, M. Alejandra Perotti

**Affiliations:** 1 Ecology and Evolutionary Biology, School of Biological Sciences, University of Reading, Whiteknights Campus, HLS Bld, Reading, RG6 6AS UK; 2 Department of Medical Molecular Laboratory, Security Forces Hospital Makkah, Makkah, Saudi Arabia; 3 Institute and Museum of Natural Sciences (IMCN), National University of San Juan (UNSJ), San Juan, Argentina

**Correction to: Experimental and Applied Acarology (2026) 96:23**

10.1007/s10493-026-01123-0

In this article, the first author’s name was incorrectly displayed as two separate authors, whereas it should have read: Riyadh Hussain S. Aeban.

Additionally, the missing online supplementary information files were added. The original article has been corrected.

## Supplementary Information

Below is the link to the electronic supplementary material.

Supplementary Material 1

Supplementary Material 2

